# Interest of a Joint Use of Two Diagnostic Tools of Burnout: Comparison between the Oldenburg Burnout Inventory and the Early Detection Tool of Burnout Completed by Physicians

**DOI:** 10.3390/ijerph181910544

**Published:** 2021-10-08

**Authors:** Céline Leclercq, Lutgart Braeckman, Pierre Firket, Audrey Babic, Isabelle Hansez

**Affiliations:** 1 Human Resources Development Unit, Faculty of Psychology and Education Sciences, University of Liège, 4000 Liège, Belgium; ihansez@uliege.be; 2 Department of Public Health and Primary Care, Faculty of Medicine and Health Sciences, Ghent University, 9000 Ghent, Belgium; Lutgart.Braeckman@UGent.be; 3 CITES ISoSL, Sector of Mental Health, 4000 Liège, Belgium; pierre.firket@me.com; 4 Group and Organization Psychology Unit, Faculty of Psychology and Education Sciences, University of Liège, 4000 Liège, Belgium; audrey.babic@uliege.be

**Keywords:** burnout, clinical judgement, OLBI, diagnosis, physicians

## Abstract

Most research on burnout is based on self-reported questionnaires. Nevertheless, as far as the clinical judgement is concerned, a lack of consensus about burnout diagnosis constitutes a risk of misdiagnosis. Hence, this study aims to assess the added value of a joint use of two tools and compare their diagnostic accuracy: (1) the early detection tool of burnout, a structured interview guide, and (2) the Oldenburg burnout inventory, a self-reported questionnaire. The interview guide was tested in 2019 by general practitioners and occupational physicians among 123 Belgian patients, who also completed the self-reported questionnaire. A receiver operating characteristic curve analysis allowed the identification of a cut-off score for the self-reported questionnaire. Diagnostic accuracy was then contrasted by a McNemar chi-squared test. The interview guide has a significantly higher sensitivity (0.76) than the self-reported questionnaire (0.70), even by comparing the self-reported questionnaires with the interviews of general practitioners and occupational physicians separately. However, both tools have a similar specificity (respectively, 0.60–0.67), except for the occupational physicians’ interviews, where the specificity (0.68) was significantly lower than the self-reported questionnaire (0.70). In conclusion, the early detection tool of burnout is more sensitive than the Oldenburg burnout inventory, but seems less specific. However, by crossing diagnoses reported by patients and by physicians, they both seem useful to support burnout diagnosis.

## 1. Introduction

Absences from work due to work-related mental disorders have strongly increased in recent years. It is now well-established that these disorders negatively impact individuals (e.g., physical and psychological health), organizations (e.g., turnover, absenteeism, lower productivity) and societies (e.g., disability costs). In Belgium, burnout is considered as a work-related disease [1]. The latest figures reached 471,040 people on long-term disability (>1 year of work disability) in 2020 (employees, unemployed, and self-employed people included) [2]. These increases can largely be explained by the mental disorder rate. Among all long-term disabilities, 78,330 people suffer from depression (16.62%) and 33,402 people suffer from burnout (7.09%) [2]. Health professionals also reported more patients expressing work-related mental disorders [3].

### 1.1. Definitions

Burnout continues not to be officially recognized within medical classifications such as the Diagnostic and Statistical Manual of Mental Disorders (DSM-V) or the International Classification of Diseases (ICD-11) [4]. These classifications do not consider burnout as a clinical diagnosis with a precise aetiology, but rather as a syndrome [5]. This can be explained by the lack of consensus concerning the definition and the dimensions of burnout. Researchers still debate the following question: should we consider burnout as a state or a process? Burnout is considered as a state by most authors, but there is a lack of consensus about the number of dimensions to include in the burnout syndrome. For instance, the Maslach Burnout Inventory (MBI), which is the main questionnaire used, consists of three dimensions [6]: emotional exhaustion, depersonalization, and reduced self-accomplishment. More recently, Schaufeli, Desart, and De Witte [7] found an alternative conception of burnout through a qualitative study among 49 health professionals. They defined it as a work-related mental state based on four main symptoms (i.e., exhaustion, mental distance, impaired emotional control, and impaired cognitive control) and three secondary symptoms (depressed mood, psychosomatic complaints, and psychological distress). A shorter definition of burnout was also advanced by Guseva Canu et al. [8] (p. 104) through a systematic review and was tested with 50 experts (researchers and health professionals aware of burnout). The proposal reached consensus among 82% of experts consulted. They defined burnout as follows: “*In a worker, occupational burnout or occupational physical AND emotional exhaustion state is an exhaustion due to prolonged exposure to work-related problems.*”

In their literature review, Hansez, Firket, and Leclercq [9] synthesized burnout as a temporal process integrating four stages. Stage 0, named *engagement and enthusiasm with a high job ideal*, is characterized by a high level of enthusiasm at work in conjunction with a high level of energy, job ambition, ideals, and ambitious work objectives. At this stage, organisational identification is high. Stage 1, entitled *the breach in the job ideal*, is characterized by contradictions and paradoxical requirements experienced at work, perceived values-based conflicts, and questioning about one’s performance. Nevertheless, further efforts, time and energy are devoted to work, until depletion of the energy reserve. *Protective withdrawal* illustrates stage 2, mainly characterized by the emergence of the first clinical symptoms and behavioral changes. Furthermore, work is no longer considered as a means of personal development. The values of the organization are rejected and workers develop coping strategies and repress their affects to protect themselves. This stage is also evidenced by repeated and ineffective sick leave and by the consequences of work-related problems extended to private life. The last stage, stage 3, named *burnout*, is usually triggered by a critical event that restores the repressed effects and may be responsible for longer-term sick leave. Burnout is characterized by a work-related identity problem, a loss of any job ideal, and a decline in work performance. This last stage is related to a feeling of shame, especially when energy for extraprofessional projects is still high. There is also a high risk of developing depressive symptoms. The work done by Hansez et al. [9] that investigates the temporal process of burnout, allows us to underline the complexity related to the diagnosis of burnout. Indeed, above burnout-related symptomatology, it is also important to consider the temporality of the process, the aetiology, individual and organizational factors, risk, and protective factors. The issue of the burnout diagnosis and its differential diagnosis definitely still requires attention in research.

### 1.2. Diagnostic Tools of Burnout

In the literature, several self-reported questionnaires (Oldenburg burnout inventory (OLBI) [10]; Spanish burnout inventory (SBI) [11]; Copenhagen burnout inventory (CBI) [12]; Maslach burnout inventory (MBI) [12]; burnout clinical subtype questionnaire (BCSQ-36) [13]; burnout measure (BM) [12]; Shirom–Melamed burnout measure (SMBM) [14]; burnout assessment tool (BAT) [7,15,16]. Note that the BAT was recently developed by Schaufeli et al. [7,15,16], based on its new dimensions. When this research was conducted, this questionnaire was still in the development and cross-validation phase in different countries and languages. In the meantime, some papers about the validation were published [7,17,18]. The Utrechtse burnout schaal (UBOS) [19]) is also reported along with its respective dimensions and psychometrics characteristics [10,20,21]. In a systematic review of 182 studies in 45 countries, Rotenstien et al. [20] identified approximately 142 definitions of burnout and at least 11 methods for measuring burnout. They showed a prevalence of burnout ranging from 0% to 80.5% among research demonstrating a high variability in the criteria of burnout. Qiao and Schaufeli [21] analyzed the convergent validity of the four most commonly used burnout self-reported questionnaires: MBI, OLBI, BM, and SMBM. They found that burnout is best assessed with a multidimensional construct and especially with two main related dimensions, namely exhaustion and withdrawal from work. The three-dimension MBI and the two-dimension OLBI reflect different ways to conceptualize the dimensions. While the MBI defines exhaustion in its affective component, the OLBI also includes exhaustion’s physical and cognitive components. Moreover, withdrawal from work is considered as depersonalization by the MBI and disengagement by the OLBI. Depersonalization reflects emotional distancing only for service providers, whereas disengagement concerns each profession and refers to distancing oneself from work and experiencing negative attitudes [10]. Regarding psychometric qualities, Sinval et al. [22] highlighted, through a meta-analysis, that most of studies showed acceptable to good internal consistency for the OLBI. Moreover, these authors found good reliability for the OLBI (Cronbach’s α = 0.93), as well as for its dimensions: exhaustion (Cronbach’s α = 0.87) and disengagement (Cronbach’s α = 0.91) among Portuguese and Brazilian samples [22]. In addition to gathering the main burnout symptoms and dimensions, the main added value of the OLBI is the balance of the items’ valence, which reduces the transparency or the social desirability bias.

Considering the high number of definitions and related self-reported questionnaires but also ongoing initiatives for new definitions and questionnaires (i.e., the BAT [7,15,16]), we can conclude that the debate about self-reported questionnaires remains unresolved. However, self-reported questionnaires have some advantages. First, subjects report their own traits or behaviors without the intervention of a third party. Second, these tools are inexpensive in terms of time and materials. Finally, manuals are often available and these allow health professionals to interpret these tools independently of their clinical experience. 

Besides self-reported questionnaires, there exist few diagnostic tools based on clinical judgement to help health professionals to establish their burnout diagnosis. Clinical judgement is a process based on the exchange of subjective and relevant information between health professionals and patients to reach clinical conclusions for an appropriate treatment [23]. It helps professionals to identify the problem in a particular context, to make a diagnosis, to consider treatment alternatives, and to predict a patient’s evolution [24]. Therefore, it requires health professionals to be able to identify and analyze the most relevant elements of the patient’s experience [24]. 

In response to these issues, the Belgian Federal Public Service of Employment, Labor, and Social Dialog [25,26] has created the early detection tool of burnout (EDTB) [27], which is intended to be completed by the health professional rather than the patient. This is the first tool to bring a structure in the clinical judgement for burnout diagnosis. The main added value of this guide is to structure the consultation and to detect the symptoms and the patterns of burnout in the patient’s discourse. In 2010, Hansez et al. [25] achieved a first version of the EDTB. This tool was pretested by general practitioners (GPs) and validated by interdisciplinary focus groups composed of 22 French-speaking and 10 Dutch-speaking health professionals (e.g., GPs, occupational physicians (OPs), psychiatrists, and psychologists). Once the GPs’ version was validated, the tool was adapted for OPs. 178 GPs and 168 OPs tested these two versions among 1089 patients. In the second study conducted in 2019 [26] the tool was updated following nine individual interviews and two focus groups, the first one composed of 11 Dutch-speaking OPs and the second one composed of 9 French-speaking OPs and 1 French-speaking GP. In total, 14 GPs and 29 OPs tested the ultimate tool among 190 patients [26]. During three months, physicians had to complete the EDTB for each patient who reported suffering at work. Then, the patient completed the self-reported OLBI questionnaire. Physicians did not have access to the results of the OLBI until they had made their diagnosis.

### 1.3. Evaluation of the Proportion of Healthy People and People Affected

Diagnostic tools have a major role in the clinician’s diagnosis to determine the presence or the absence of burnout (binary diagnosis). Diagnostic accuracy is represented by a two-by-two table. Hence, when a test provides metric results, it can be useful to establish or change the cut-off score to evaluate the test’s validity, which is the ability of the test to classify disease and healthy subjects according to a reliable reference method. For an accurate diagnosis, we need to evaluate the rate of cases with and without burnout through sensitivity (SE) and specificity (SP) [28,29]. As defined by Hajian-Tilaki [29] (p. 2374), sensitivity reflects “*the proportion of test positivity given the presence of a target condition*” and specificity is “*the proportion of those who are disease-free and who are labelled negative by the diagnostic test*”. Therefore, sensitivity represents the ratio of true positives and specificity integrates the ratio of true negatives. Sensitivity will be equal to 1 when the test diagnoses all diseases and to 0 when it detects none. In the same way, when a negative result corresponds to all people without the disease, specificity will be 1. We can choose either a high sensitivity to exclude burnout for healthy people or a high specificity to diagnose burnout for people affected by burnout [30]. The strategy depends on the cost–benefit ratio, and moderate results can be acceptable for screening burnout to favor a low false-negative rate [30]. 

Two other parameters are also used to evaluate the probability of being affected or not by burnout depending on test results. These are the positive predictive value and the negative predictive value, which depend on the prevalence of the disease. As defined by Hajian-Tilaki [29] (p. 2374–2375), the positive predictive value is “*the proportion of presence of target condition given a positive test result*” and the negative predictive value is “*the proportion of being healthy among those with negative test results.*” 

### 1.4. The Comparison and the Joint Use of Diagnostic Tools

As seen in the literature, increasingly more studies are focused on comparison and the joint use of different tools to support diagnosis in medical and psychological fields, such as human health and behavior [23,24,25,26,27,28,29,30,31], sex offenders [32], frailty among elderly [33], hyperdentinal sensitivity [34], and burnout [35,36,37]. Using various methods, researchers reported divergent results regarding the contribution of a joint use of clinical judgement and assessment tools. Some results concluded that tests outperform or perform at least as well as clinical judgement [23,31,32,33]. Others concluded that clinical judgement has a better performance in supporting the diagnosis [34,35,36]. Nevertheless, some authors agreed to include a self-reported questionnaire or to jointly use different assessment tools to structure the clinical judgement in order to improve the diagnosis [32,34,35,37].

Grove et al. [23] conducted a meta-analysis to compare the accuracy of clinical judgement (e.g., informal and subjective methods) and mechanical prediction. They defined mechanical prediction as statistical, actuarial, and algorithmic predictions that can be fully reproducible and do not require expert interpretation [23]. Their meta-analysis included 136 psychological and medical studies comparing the performance of clinical judgement and mechanical prediction. Studies involving nonhuman research were excluded. Results showed that mechanical prediction was superior in 63 studies and equal in 65 studies. Only eight studies demonstrated better performance of clinical judgement, mainly explained by more data available for the clinicians. This meta-analysis showed that the accuracy of mechanical prediction is generally superior or at least equal to clinical judgement. Some factors reduce the clinicians’ performance such as the lack of data availability, heuristics and biases, unknown rates and statistical cues, and inadequate feedbacks. These results are supported by Ægisdóttir et al. [31] through a similar meta-analysis among 67 studies that compared mechanical and clinical predictions. They found 92 effect sizes, and the overall effect highlighted the accuracy of the mechanical prediction. They extracted 48 effect sizes among the strictest studies and identified mechanical prediction as 13% more accurate than clinical prediction. 

If we consider now specific studies in the medical and psychological fields, Van Vugt et al. [32] compared two methods to assess victim empathy among 85 young male offenders. The basic empathy scale (BES) was compared with the clinical judgement of victim empathy. The BES is a validated self-reported questionnaire assessing the subject’s cognitive and affective empathy with a five-point Likert scale, and the clinical judgement was a threefold diagnosis (victim empathy is present, slightly present, or is lacking). As for the moral sense of young male offenders, they compared mean scores with the three diagnoses, but they found significant differences in the means score of the BES for the three clinical judgement categories. However, the clinical judgement categories did not match with the BES results (e.g., the category “victim empathy is slightly present” had a lower score on the BES than the category “victim empathy is lacking”). Although they did not show that unstructured clinical judgement yielded invalid information, they pointed out that structured judgement based on multiple sources of objective assessment tools outweighed the risks of therapeutic biases and distorted clinical judgement. Moreover, clinical judgement remains crucial for contextualizing and interpreting relevant information. 

These findings are in line with the results of Kirkhus et al. [33] who compared oncologists’ clinical diagnosis of frailty with the self-reported “modified geriatric assessment (mGA)” among 307 older cancer patients. To compare the two diagnoses, the authors transformed the threefold classification of the oncologists’ diagnoses into binary diagnoses by gathering moderately and severely ill patients. The mGA allowed the identification of 139 patients as frail and 149 patients as non-frail, whereas the oncologists’ clinical judgement identified106 patients as frail, and 182 patients as non-frail. Even clustering moderate and severely ill patients, the clinical judgement made by oncologists still missed 67 frail patients according to the mGA. The authors concluded that it is preferable to systematically include the mGA in the diagnosis. 

Conversely, Barroso et al. [34] conducted a cross-sectional study to compare a self-reported questionnaire relating to hypersensitive teeth with clinical judgement. Among the 380 Brazilian participants, 158 reported a presence of dentinal hypersensitivity (DN) in a self-reported questionnaire with one question about the presence or the absence of DN, whereas 336 were diagnosed with DN by clinical judgement. However, they also evaluated the accuracy of the cold water and tactile tests and obtained a receiver operating characteristic (ROC) curve with near-perfect accuracy (99%) for both tests. There was a significant underestimation of the prevalence of DN in the self-reported questionnaire. The joint use of different tools was recommended for the DN diagnosis because in line with psychological issues, there is a subjective experience that affects the perception of the patient.

In the context of work-related stress, Schaufeli et al. [35] explored the validity of the MBI [6,7,8,9,10,11,12] and the BM [12] in a sample of 139 employees who sought psychotherapeutic treatment. They used a work-related form of neurasthenia from the International Classification of Diseases criteria (ICD-10) as a reference method of clinical burnout to validate the MBI and the BM. Among their sample, according to the ICD-10, 71 employees were affected by burnout, whereas other patients were diagnosed with other mental disorders. The three-factor model of the MBI (i.e., emotional exhaustion, depersonalization, and reduced self-accomplishment) was validated in the clinical sample. Their findings did not include concrete cut-off scores, but they found a sensitivity of 70% and a specificity of 57% for the MBI. They concluded that the MBI can distinguish 70% of people with burnout and 57% of people without burnout. They compared the MBI with the BM, which is less sensitive (60%) but more specific (71%) than MBI. 

In 2013, Kleijweg et al. [36] replicated the study of Schaufeli et al. [35]. They administered the MBI and the mini international neuropsychiatric interview (MINI), a semi-structured interview based on classifications in the DSM-IV, to 439 Dutch patients from an occupational clinic that specialized in work-related psychological problems. They compared the MBI scores with the diagnosis resulting from the MINI. Through a ROC curve, they explored different cut-off scores to improve the discriminant validity of the MBI but did not find a sufficiently discriminant cut-off score. However, results showed an optimal cut-off score of 3.50 on the exhaustion subscale, with a sensitivity of 78% and a specificity of 48%. This means that the MBI probably overdiagnoses burnout. Contrary to Schaufeli et al. [35], Kleijweg et al. [36] concluded that the MBI has a poor discriminant validity for clinical use and recommended using the cut-off score of 3.5 for the exhaustion subscale ifused. 

However, Wickramasinghe et al. [37] also pointed out that research progress on burnout is limited due to the lack of cut-off scores for a dichotomous diagnosis. They found cut-off scores for the MBI student survey (MBI-SS) with a clinical correlation study. Among 194 students in Sri Lanka, clinically validated cut-off scores were developed by using the clinical diagnosis of the consultant psychiatrist as the reference method. Through a ROC curve, they found cut-off scores of 12.5 for emotional exhaustion, 7.5 for cynicism, and 10.5 for reduced professional efficacy, and determined that the test could be used as a burnout screening tool [37]. The Sinhala translation of the MBI-SS showed good accuracy with a sensitivity of 91.9% and a specificity of 93.2%. As shown by Schaufeli et al. [35] and Wickramasinghe et al. [37], clinical validity of the MBI can be verified among clinical patients and can support scientific validity.

The following table (Table 1) resumes the main results of these three burnout studies:

Some authors [35,36,37] compared the MBI, the BM, and the MBI-SS to a structured clinical judgement as the reference method. Note that they did not use the self-reported measure as a reference method. Consequently, we found it interesting to go one step further in this study by considering both the clinical judgement and the self-reported questionnaire as reference methods. Therefore, based on the above, the following research question is deconstructed into three hypotheses:

“What is the added value of jointly integrating a self-reported questionnaire and an interview guide in the burnout diagnosis?”

First, we postulate that the sensitivities and the specificities of the OLBI and the EDTB allow discrimination between healthy people and people affected by burnout.

**Hypothesis** **1** **(H1)**. *The OLBI and the EDTB are each able to reasonably distinguish healthy people (specificity) and those suffering from burnout (sensitivity).*

Some of the research findings showed that statistical methods tend to outperform clinical judgement when the clinical judgement is only based on the health professional’s skills [23,31,32,33]. However, they also pointed out that clinical judgement can outperform or perform as well as the statistical methods when the health professionals have more data available to structure the clinical judgement. Based on contradictions from the literature, we postulate that the clinical judgement has a similar or a better performance than the OLBI when it is structured by the EDTB.

**Hypothesis** **2** **(H2)**. *Clinical judgement structured by the EDTB outperforms or performs at least as well as the OLBI.*

Furthermore, many health professionals are involved in the treatment of patients suffering from burnout, such as GPs, OPs, psychiatrists, clinical or occupational psychologists, or psychosocial prevention counsellors. The focus on the burnout analysis and the diagnosis could be different according to the type of health professional. For example, in psychology, Evans et al. [38] highlighted differences between psychologists’ perspectives on the classification of mental disorders according to the clinical experience, the roles, the training background, the diagnostic practices, the classification systems used, the culture, and so on. Hence, we postulate that the EDTB structured and homogenized the clinical judgement among physicians by comparison with the OLBI.

**Hypothesis** **3** **(H3)**. *The clinical judgement structured and homogenized by the EDTB outperforms or performs at least as well as the OLBI regardless of the type of physician who makes the diagnosis.*

## 2. Materials and Methods

### 2.1. Objective

This study aims to verify and compare the accuracy of the OLBI and the health professionals’ clinical judgement structured by the EDTB and to highlight the interest of joining both tools to support burnout diagnosis.

### 2.2. Procedure

The data from this study comes from research previously conducted to assess the prevalence of burnout within the Belgian working population and the interest of joining two diagnostic tools of burnout [26]. The Ethics Committee of the Faculty of Psychology, Speech Therapy, and Educational Sciences of the University of Liège (ULiège) authorized this study (ID: 1920-94). For each consultation with a patient who reported suffering at work, physicians asked the patient’s consent to include him or her in the study. Once agreement was obtained, physicians had to complete the EDTB online, either during or right after the consultation. In addition to this clinical judgement tool, physicians asked each patient to complete a paper version of the OLBI. Patients could complete this questionnaire after the consultation, either in the waiting room or at home, and return it to the physician. The physician completed the EDTB before receiving the results of the OLBI. In order to link the EDTB to the OLBI, physicians assigned a participation number to each patient and associated this number with the two completed tools. Once a month, physicians returned questionnaires to researchers without any identifying information. This paper reports the results of this cross-sectional study in such a manner as Barroso et al. [34], which provides data at a specific point in time.

### 2.3. Participants

The target population concerns people who have consulted a GP or an OP and who have expressed complaints and symptoms of suffering at work. Moreover, we only included patients within our sample for whom we were able to link the clinical judgement done by either the GP or the OP with the EDTB, to the results obtained from the OLBI. In total, our sample was composed of 123 patients (Table 2).

### 2.4. Measures

#### 2.4.1. Oldenburg Burnout Inventory (OLBI)

Burnout was measured using the validated French and Dutch versions of the OLBI [10]. The OLBI is a self-reported measure of 16 items evaluating burnout independently of work context, with the job demands–resources model [39] as theoretical background. The OLBI presents good psychometric properties [10,20,21]. The OLBI evaluates burnout using two dimensions: (1) exhaustion with eight items (e.g., ‘After work, I regularly feel worn out and weary’), and (2) disengagement with eight items (e.g., ‘I frequently talk about my work in a negative way’). Exhaustion represents the consequence of physical, emotional, and cognitive efforts [10]. The disengagement dimension is essential to distinguish burnout from chronic fatigue [10]. Disengagement refers to distancing oneself from work and a negative attitude towards others [10]. Participants were asked to respond by using a four-point Likert scale ranging from 1 (strongly disagree) to 4 (strongly agree). Responses are coded in such a way that a high score corresponds to a high level of exhaustion and/or disengagement. This questionnaire is balanced as far as positive and negative wording of the items is concerned. It allows the person to think carefully about the content of each item [21] and suggests that burnout is a process illustrated by a continuum between exhaustion and vigor, and by a continuum between cynicism and dedication [10].

#### 2.4.2. Early Detection Tool of Burnout (EDTB)

The EDTB was developed within the framework of research funded by the FPS Employment, Labor and Social Dialog [25,26]. This tool is available in French and Dutch versions, but also in two slightly different versions, one dedicated to GPs and another to OPs [27]. For both versions, the EDTB is a three-page document integrating the following themes: reported general complaints (e.g., disturbed sleep, stress, workload, conflict at work), burnout symptoms (physical, cognitive, affective, and behavioral), work-related aspects (origin of complaints, risk factors, job demands, and lack of job resources), socio-demographic characteristics, absenteeism information, diagnosis (e.g., burnout, anxiety, and depression), and additional comments. Moreover, specifically in the OPs version, two additional items are included: type of medical examination and clinical conclusion, raising the question of functional capacity to work. For each theme, health professionals can tick one or more elements corresponding to the worker’s reported experience. In this study, the final diagnosis reported by the health professional ‘i.e., presence or absence of burnout’, is the main information used. 

### 2.5. Analyses

In this study, we first identified the validity of the EDTB and the OLBI by taking turns to consider respectively the OLBI and the EDTB as the reference method. Second, we compared the test validity indicators (i.e., sensibility, specificity, positive and negative predictive value, precision) between the two tools.

First, the EDTB allows a binary diagnosis, either the presence of burnout (i.e., a positive result) or the absence of burnout (i.e., a negative result). 

Second, OLBI scores range from 16 to 64 and can be converted into a threefold classification: low (scores below 30), medium (scores between 30 and 45), or high (scores above 45) according to Hansez and Laurent [40]. In order to match with a double entry table as recommended in the literature, we decided to establish a cut-off score to define either a positive or a negative score for the self-reported questionnaire. 

According to Hajian-Tilaki [29], psychometric qualities depend on the cut-off score chosen. They recommended using validated methods for identifying the cut-off score, such as the receiver operating characteristics (ROC) curves. 

In this study, we focus on the ROC curve, which is the plot of sensitivity versus 1—specificity. This curve provides an optimal cut-off score to distinguish people suffering from burnout from healthy ones, and to evaluate the test performance [29]. It shows all possible cut-off scores for a continuous or an ordinal scale. The several points, which can be represented on a graph, correspond to all cut-off scores that can be used to determine whether the test results are positive. The area under the ROC curve (AUC) evaluates the contribution test to the diagnosis as a continuum between useless information (AUC = 0.5) to very useful information (AUC = 1). The more the AUC tends towards 1 (100% true positives), the more the test is considered to be discriminating and its results as reliable [29]. Moreover, the AUC also refers to the likelihood that the burnt-out person will score higher than the healthy person’s score. 

According to our first hypothesis (H1), comparison analyses between EDTB and OLBI were carried out using R software [41]. Based on two cross-tables (Table 3 and Table 4), we calculated sensitivity (i.e., the probability of burnout for positive results), specificity (i.e., the probability of being healthy for negative results), positive predictive value (i.e., the probability of burnout for positive results), negative predictive value (i.e., the probability of being healthy for negative results), and the overall level of agreement with the Cohen’s kappa. In Table 3, we evaluated the validity of the OLBI based on the clinical judgement as the reference method. In Table 4, we assessed the validity of the clinical judgement based on the OLBI as the reference method.

Finally, McNemar’s chi-squared analysis was used to compare the validity of the OLBI and the EDTB (H2). We also used Fisher’s exact test to compare the validity of the clinical judgement (EDTB) between GPs and OPs (H3).

## 3. Results

### 3.1. Cut-Off Score for the OLBI

**Figure 1:**
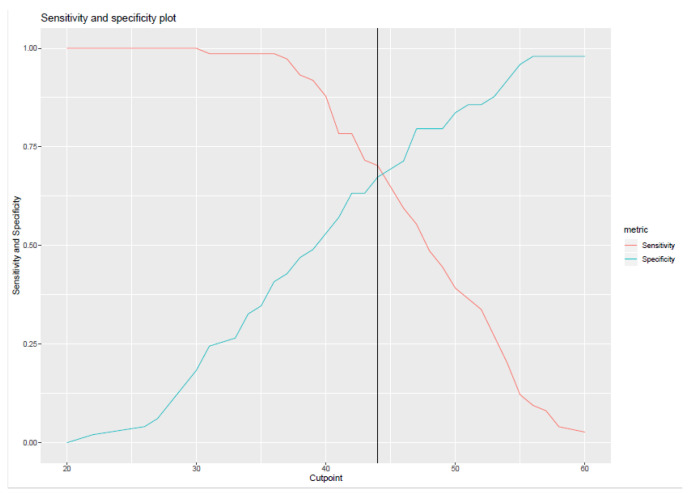
Identification of the cut-off point by crossing sensitivity and specificity.

**Figure 2:**
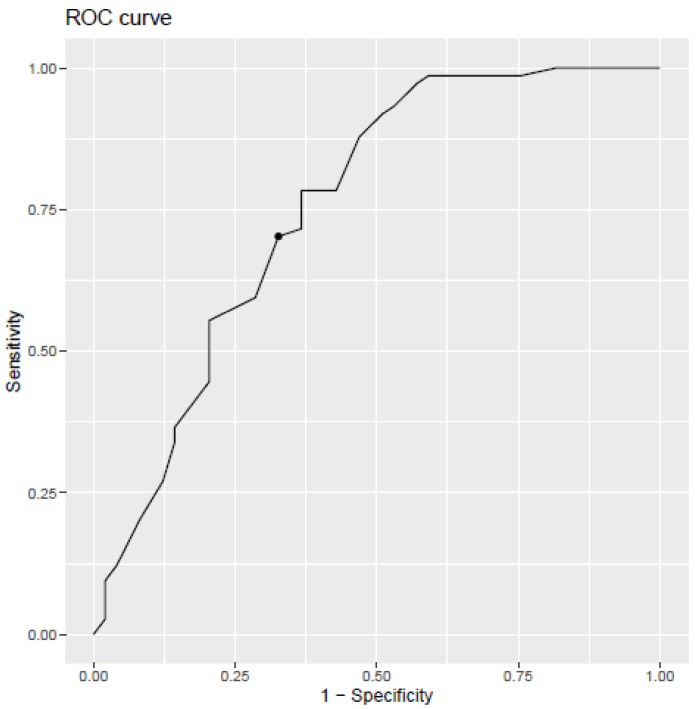
Distribution of cut-off scores for the Oldenburg burnout inventory (OLBI).

As seen in Figure 1, the ROC curve highlighted a cut-off score of 44 for the self-reported questionnaire with a sensitivity of 70.27% and a specificity of 67.34%. This means that all scores below 44 are considered as negative (absence of burnout), while scores equal to or above 44 are considered as positive (presence of burnout). For a test with a perfect discrimination between true positive and true negative cases, the sensitivity and specificity should be 100%. With a sensitivity of 70.27% and a specificity of 67.34%, the area under the curve (AUC) is 0.754 (95% confidence interval 0.662–0.846), as seen in Figure 2. 

### 3.2. Comparison between the OLBI and the EDTB

Based on this new cut-off score, 68 patients had a positive burnout diagnosis (55.28%) and 55 patients (44.72%) had a negative burnout diagnosis according to the OLBI. According to the binary classification of the EDTB, physicians identify 74 cases of burnout, compared to 49 cases of non-burnout. By crossing the scores obtained on the OLBI and on the EDTB, we obtained four groups (Gr) in Table 5. In 69.10% of cases, both tools reach the same conclusion, either presence of burnout (Gr1) or absence of burnout (Gr4). Nevertheless, results diverge in 30.90% of cases. For 22 patients, the physician diagnoses burnout, whereas the OLBI’s results highlight an absence of burnout (Gr2). Conversely, 16 patients diagnosed with burnout by the OLBI were not recognized as suffering from burnout according to the EDTB (Gr3). 

Since we do not know the actual state of the patient, we carried out two analyses to compare the tools.

The first analysis was done by considering that the OLBI reflects the actual state of the patient. The reference method corresponds to the OLBI and the method tested corresponds to the EDTB. In the second analysis, we consider that the EDTB reflects the actual state of the patient. The reference method corresponds to the EDTB and the method tested corresponds to the OLBI.

As can be seen in Table 6, the EDTB detects 76% true positives, while the OLBI detects 70% of burnout cases. However, the specificity of the OLBI is higher (67%) than the EDTB (60%). The OLBI excludes more people who do not suffer from burnout. Results confirm our first hypothesis (H1) postulating that the OLBI and the EDTB are each able to reasonably distinguish healthy people (specificity) and those suffering from burnout (sensitivity). Using the 44 OLBI cut-off score, we found a fair overall rate of agreement with the structured guide interview (EDTB), with a significant but fair kappa (α = 0.36, *p*-value < 0.001). 

In clinical practice, health professionals must primarily consider two other settings, which are the positive predictive value (PPV) and the negative predictive value (NPV) (Table 6). These values provide information on the probability of burnout if the test is positive, and on the absence of burnout if the test is negative. In any hypothetical population, the probability of a person being diagnosed positive is 70% for the EDTB, and 76% for the OLBI. For the negative predictive value, a person with a negative diagnosis has 67% chance of not being diagnosed with burnout for the EDTB, and a 60% chance for the OLBI. The accuracy is the ability of the test to generate a score closest to the score of the reference state. For both analyses, accuracy is 69%. Both tools reach the same conclusion in 69% of cases.

Applying McNemar’s chi-squared test, we noticed a statistically significant difference between sensitivities in favour of the clinical judgement (70% for the OLBI versus 76% for the EDTB; Chi-squared = 18.02, *p*-value < 0.001). However, we did not detect a significant difference between specificities (67% for the OLBI versus 60% for the EDTB; Chi-squared = 1.82, *p*-value = 0.18). These results confirm our second hypothesis (H2), postulating that the clinical judgement structured by the EDTB outperforms, or performs at least as well as the OLBI.

### 3.3. Comparison of the Clinical Judgement Made by General Practitioners (GPS) and Occupational Physicians (Ops) with the OLBI

Forty-three physicians, including 14 GPs and 29 OPs, participated in the study. In our sample (*N* = 123), 100 patients consulted an OP and 23 consulted a GP. Of these, 54 patients were diagnosed as suffering from burnout and 46 were considered to be healthy by OPs (Table 7), while GPs diagnosed burnout for 20 patients out of 23 (Table 8).

We compared both tools amongst OPs and GPs (Table 9). We observed significant differences between sensitivities (Chi-squared = 10.87, *p*-value = 0.001) and between specificities (Chi-squared = 5.45, *p*-value = 0.02) for occupational physicians, whereas we only found a significant difference between sensitivities (Chi-squared = 7.56, *p*-value = 0.01) for general practitioners (difference between specificities was not significant, Chi-squared = 2.29, *p*-value = 0.13). These results partially confirm our third hypothesis, that the clinical judgement structured and homogenized by the EDTB outperforms or performs at least as well as the OLBI, regardless of the type of physician who makes the diagnosis.

## 4. Discussion

The aim of this study was to highlight the interest of a joint use of two diagnostic tools for burnout: the EDTB and the OLBI.

The first hypothesis concerns the discriminating power of the OLBI and the EDTB (H1). Among the 123 patients, we found a cut-off score of 44 for the OLBI with a sensitivity of 70.27% and a specificity of 67.34%. With a sensitivity of 70.27% and a specificity of 67.34%, the area under the curve (AUC) is 0.754 (95% confidence interval 0.662–0.846), as seen in Figure 2. The area under the curve assesses the probability that the OLBI score should be higher for patients diagnosed with burnout by the physician. This means that, in 75.4% of cases, the person diagnosed with burnout by the physician will have a higher score than the person diagnosed not to have burnout by the physician. According to Yang and Berdine [42], the test has a good contribution to diagnosis (0.7 ≤ AUC < 0.8). However, using the cut-off score of 44 for the OLBI, there is a fair overall rate of agreement with the structured interview guide (EDTB). Although the overall agreement is considered as fair (0.36), it is significantly different from the diagnosis obtained randomly [43]. Analyses show that the EDTB detects 76% of people with burnout, highlighting a slightly higher sensitivity than the OLBI, which identifies 70%. Moreover, the new cut-off score preserves good specificity for each of the tools. The specificity of the EDTB makes a negative diagnosis for 60% of people without burnout. This is lower than the OLBI, which detects 67% of unaffected people. To summarize, the EDTB seems to be more sensitive but less specific than the OLBI. According to Trevethan [30], a diagnosis rarely confirms or invalidates with certainty the presence or absence of a disease. A test with high sensitivity aims to exclude the disease for people with a negative test by reducing the number of false negatives. It means that people with a negative result are very unlikely to have the disease. Inversely, a test with a high specificity aims to diagnose the disease for people with a positive test by reducing the number of false positives. In other words, people with a positive result are certainly affected by the disease. With sensitivity higher than 70% and specificity higher than 60%, we can reasonably exclude a burnout diagnosis for healthy people, while diagnosing a maximum of people suffering from burnout. According to Power, Fell, and Wright [44], a test is considered as useful when the sum of the sensitivity and the specificity is around 1.5 (1 is useless and 2 is perfect). Our results showed 1.36 for the EDTB and 1.37 for the OLBI. Although it is lower than 1.5, it is still acceptable concerning the small sample size [44]. Therefore, we can conclude that the OLBI and EDTB are both able to reasonably discriminate healthy people from people affected by burnout in this Belgian context. Moreover, the discriminant power of both tools is higher than in previous studies based on other OLBI cut-off points [25,26].

Compared with the MBI tested by Schaufeli et al. [35] and Kleijweg et al. [36], this study highlights the fact that the OLBI has a similar, or a slightly lower sensitivity than the MBI, but a higher specificity. While Schaufeli et al. [35] concluded that the MBI discriminates between burnt-out and healthy people with findings similar to ours, Kleijweg et al. [36] concluded that there was poor discriminant validity for the MBI, due to low specificity that emphasizes a risk of overdiagnosing burnout. In 2018, Wickramasinghe et al. [37] found a cut-off score for a dichotomous diagnosis of the MBI-SS and obtained an almost perfect sensibility (0.91) and specificity (0.93). In line with Schaufeli et al. [35] and Wickramasinghe et al. [37], we found good discriminant power with respect to the self-reported questionnaire, the OLBI. These results support the use of a score cut-off to increase the discriminant power and the importance of using self-reported questionnaires in the burnout diagnosis. According to Shoman et al. [12], OLBI is the second most valid available burnout self-reported questionnaire. Moreover, the latest findings on self-reported questionnaires [25,26,35,36,37] and the results from this study support the clinical use of self-reported questionnaires in various countries (The Netherlands, Sri Lanka, and Belgium). Other studies in different countries and among various populations focused on the advantages of using self-reported questionnaires. For example, Sinval et al. [22] concluded that the OLBI is relevant to compare burnout among countries based on two general samples in Brazil and Portugal. On the African continent, the OLBI was also considered as useful, for example, to identify characteristics of the burnout syndrome among nurses [45]. 

Relating to the structured interview guide, there is no study on the EDTB in Belgium, except studies on its creation [25,26,27]. However, another study in Switzerland tests the diagnostic performance of the EDTB and compares it with the OLBI. The authors suggest that the EDTB is useful to identify moderate and proven burnout in the Swiss context [46,47].

According to the second hypothesis (H2) concerning the difference between the sensitivity and the specificity of both tools, we found a significant difference for sensitivities, but not for specificities. Hence, our second hypothesis is validated. Unlike Grove et al. [23], Ægisdóttir et al. [31], van Vugt et al. [32] and Kirkhus et al. [33], we concluded that the clinical judgement made by the EDTB has better sensitivity than the OLBI, and performs as well as the OLBI for the specificity. 

According to Grove et al. [23], clinical judgement needs to have more data available to outperform or perform as well as the mechanical prediction (e.g., self-reported questionnaire). Our study showed that clinical judgement structured by the EDTB gives health professionals more information/data to establish a better diagnosis and this finding supports the benefits of a complementary approach that the joint use of different tools can offer. Based on similar findings, Van Vugt et al. [32] and Kirkhus et al. [33] recommended including multiple sources of objective assessment tools to structure the clinical judgement and to offset biases. These results support the general use of different tools to structure clinical judgement and to bring more data to the clinical practice. Nevertheless, Barroso et al. [34] pointed out a specific caution for self-reported questionnaires. They recommended combining self-reporting with other tools, due to the subjective experience of the patient. In our study, the EDTB based on the health professional’s judgement can reasonably offset the biases of the patient’s perception. 

In order to evaluate our third hypothesis postulating that the clinical judgement structured and homogenized by the EDTB outperforms, or performs at least as well as the OLBI regardless of the type of physician who makes the diagnosis, we compared the OPs and GPs’ clinical judgement with the OLBI. We found significant differences between sensitivities and between specificities for OPs, and we found a significant difference between sensitivities for GPs. Thus, it partially confirms our third hypothesis (H3). Indeed, the clinical judgement structured by the EDTB outperforms or performs as well as the OLBI to detect people suffering from burnout among both types of physicians. These results are particularly relevant for GPs, and more moderate for OPs. However, the EDTB completed by OPs seems to slightly underperform in detecting healthy people. This could be explained by the focus on work difficulties rather than on the differential diagnosis. The small specificities for the comparison between GPs and OLBI can be explained by the small sample size of 23 patients. Moreover, 14 patients were diagnosed with burnout by both tools, 8 obtained contradictory results, and 1 was diagnosed as healthy by both tools. 

Furthermore, it is also interesting to take into consideration contradictory diagnoses. This provides information about social desirability bias, which can have an impact on the symptoms reported to physicians or during the completion of the OLBI, and thus generate contradictory results between the clinical judgement and the OLBI. However, it highlighted the need to deepen clinical judgement by using other tools to confirm the diagnosis of burnout or consider other disorders such as depression, stress, anxiety, chronic fatigue, etc. In this study, 16 patients who were diagnosed with burnout by the OLBI were not recognized as suffering from burnout by the physician, and 22 patients obtained the reverse results. These divergent results illustrating the complexity of the burnout diagnosis can be explained by the lack of consensus regarding the classification of symptoms related to burnout [20]. Another reason, to explain a non-burnout clinical judgement for a high OLBI score, could be the difficulty for practitioners in this field to put a label of burnout on a patient. This is why some physicians diagnosed others mental disorders such as depression, but also life/work difficulties; early burnout, or being at risk of burnout comorbidities between stress, burnout and depression; anxiety or chronic PTSD [4,27].

According to each diagnostic tool, what is the probability that people with a positive diagnosis truly have the disease? What is the probability that people with a negative diagnosis truly do not have the disease? These questions reflect the positive and negative predictive values. According to our results, in a theoretical population, the probability of being affected by burnout is 70% for the EDTB and 76% for the OLBI, and the probability of not being affected by burnout is 67% for the EDTB and 60% for the OLBI. Nonetheless, these cues depend on the prevalence of the disease in the population [30]. A positive test is more likely to be a false positive, if the prevalence of the disease is low. This component can constitute a limit because we do not know the actual burnout prevalence, which ranges from 0% to 80.5% according to Rotenstein et al. [20], in particular, due to the lack of consensus about its definition and diagnosis. This limit regarding the prevalence of burnout further highlights the added value of using different measurement tools to support and homogenize the diagnosis. 

Based on Trevethan [30], it is better to obtain a high PPV to minimize false positives when risks and costs are high (e.g., financial costs of treatment or risk of overtreatment) and a high NPV to minimize false negatives when the benefits of treatment are high [30]. However, moderate results are acceptable when risks and costs associated with the treatment are minimal [30]. For burnout, we consider that there may be more risks associated with having no psychological treatment program. At the individual level, there are several consequences on physical and psychological health. For instance, burnout can lead to depression, which implies strong medication and hospitalizations [48]. At the organizational level, it implies more absenteeism, a risk of turnover, and so on. At the societal level, stress and burnout are currently responsible for a third of the total number of days of sickness absence in Belgium. According to the National Institute for Health and Disability Insurance [2], the number of people (employed, unemployed, and self-employed) compensated due to long-term incapacity for work is estimated at 471,040 people in 2020. Among all long-term disabilities, 7% were due to burnout (33,402 people) and 16.6% due to depression (78,330 people) [2]. The total compensation for these people reached €6.6 billion in 2019 and more than €1.5 billion is allocated for long-term disabilities due to burnout and depression [2]. These figures highlight the high costs associated with long-term disabilities due to mental disorders at work.

By considering burnout as a process that evolves over time [9], it is possible to define different perspectives of prevention: primary, secondary, and tertiary prevention [9]. Indeed, even in a diagnosis of absence of burnout, people with work-related risk factors may take advantage of primary prevention such as working conditions improvement or adaptation. However, the human and financial costs associated with secondary and tertiary prevention are higher. A well-established diagnosis helps to reduce the costs associated with inadequate treatment. Hence, health professionals need to focus on the diagnosis before proposing an adapted treatment. For instance, in Belgium, Fedris (Federal Agency for Occupational Risks) started in 2019 a pilot-project: a treatment program for workers suffering from burnout at an early stage [49]. Practically, they recommend jointly using the EDTB and the OLBI in clinical judgement in order to only include in the program workers suffering from burnout at an early stage, and to reorient those who need an alternative treatment.

### Limitations, Recommendations for Future Research, and Practical Implications

This study contributes to the advancement of knowledge in the area of the diagnosis of burnout by purposing a combined methodology based on the patient and the health professional’s perspective to improve the burnout diagnosis. The clinical judgement and the clinical validity of self-reported questionnaires are relatively unexplored in the burnout literature and require more investigations. Our research contributes to fill this gap.

The first limitation concerns the sample size (*N* = 123), which limits the generalizability of our findings. Therefore, results must be interpreted with caution. However, results showed that both tools have good validity and could reduce the risk of misdiagnosis. Concerning the physicians, GPs are poorly represented, with only 14 GPs. This could be explained by the fact that GPs have fewer work-related consultations. Moreover, this study was conducted in a Belgian context. García-Arroyo and Segovia [50] showed differences in burnout intensity between Latin American countries, mainly related to occupation and language. This study also involves a sociocultural limitation due to the small sample size, the Belgian context, and the health professionals involved.

The second limitation concerns the prevalence of burnout in the Belgian population, which is essential to properly interpret the predictive values (PPV and NPV). It is difficult to obtain such indicators because of the lack of consensus about the definition and the diagnosis. For example, Hansez et al. [25] assessed the prevalence of burnout among 135,131 contacts reported by GPs or OPs. The physicians identified 1089 cases of burnout in the sample (0.8% burnout cases). Moreover, results are difficult to compare notably due to cultural differences or methodology. According to the variability in the prevalence of burnout currently, we cannot rely on a specific prevalence. The burnout prevalence definitely still requires standardization through a consensus concerning the definition and the diagnostic tools.

The third limitation relates to the validation of the EDTB. This tool is not yet validated or published. However, a recent study is evaluating the tool’s efficiency for the diagnosis of burnout [46,47]. The authors posit that the EDTB is useful to identify moderate cases and prove burnout in both the Belgian and Swiss contexts. However, the tool is not very suitable in its current form and needs to be adapted to the Swiss context and an appropriate training for health professionals can be useful to adjust the EDTB in the field [46,47]. 

The fourth limitation concerns the single-level comparison. This study includes a risk of social desirability bias. Nevertheless, this risk seems minimal considering the range of diagnoses for both clinical judgement and OLBI. Furthermore, the clinical judgement is only based on the EDTB. 

The fifth limitation concerns the level of agreement between the OLBI and the EDTB. There is a fair overall level of agreement. However, even if the level of agreement is fair, this is higher than diagnosis obtained by random. This limitation could be explained by the nature of both tests, one is completed by the patient, and the other by the health professional. 

Based on each limitation, we propose some recommendations for future studies. First, it would be interesting to replicate the comparison between self-reported questionnaires and clinical judgement in other countries based on a structured interview guide, such as in the Swiss study [46,47]. In line with the second limitation, and considering the increase in burnout-related disabilities in recent years, it would be interesting to assess the prevalence of burnout in Belgium, such as Hansez et al. in 2010 [25]. In response to the third limitation, the EDTB needs to be tested in other countries where burnout management may be provided by other health professionals, and where collaboration with other health professionals is different. According to the fourth limitation, further research could focus on four group comparisons. It would allow comparing the variability of health professionals’ clinical judgement without any tools (group control) with three conditions, namely health professionals’ clinical judgement made only with the EDTB; or made only with the OLBI; and then made with both tools. This method aims to compare the added value of one tool (the EDTB or the OLBI) with the one of both these tools together. This method could also be used to compare these diagnoses with other burnout tools. 

## 5. Conclusions

Both the OLBI and the EDTB have good clinical and intrinsic validity. The EDTB is more sensitive than the OLBI, but tends to be less specific. Moreover, in a hypothetical population, clinical judgement made with the EDTB and diagnosis made with the OLBI predict respectively that 70–76% of people diagnosed with burnout would be affected by burnout, and 60–67% of people diagnosed as being healthy by both diagnoses would really be healthy. According to our results, each tool provides clarifications about the burnout state by crossing diagnoses reported by patients and by physicians. Furthermore, the balance between sensitivity and specificity is a strategic choice for health professionals, which takes into consideration risks and benefits of beginning a specific psychological treatment for burnout, or reorienting the patient if the differential diagnosis leads to another mental disorder. As discussed in this paper, many health professionals with different burnout sensibilities are involved in the treatment. Hence, these findings emphasize the importance of developing a consensus about the burnout definition, standardizing and combining these two diagnostic tools (EDTB and OLBI), and implementing specific trainings based on exchanges between health professionals about clinical cases (e.g., intervision group or supervision).

## Figures and Tables

**Table 1:** Synthesis of main results among burnout studies.

References	Population	Reference Method	Tested Method	Test Validity of the Tested Method	
				Sensitivity	Specificity
Schaufeli et al. [35]	Netherlands workers	Clinical judgement using the ICD-10 diagnostic guidelines	MBI	0.70	0.57
			BM	0.60	0.71
Kleijweg et al. [36]	Netherlands workers	Semi-structured interview with the mini international neuropsychiatric interview	MBI	0.78	0.48
Wickramasinghe et al. [37]	Sri Lankan students	Clinical judgement made by a consultant psychiatrist	MBI-SS	0.92	0.93

**Table 2:** Subject demographics, *N* = 123.

**Age**, mean, years (SEM *)	44.47 (+/− 10.13)	**Job tenure**, mean, years (SEM)	15.53 (+/− 10.93)
**Genre**, *N*		**Contract**, *N*	
Men	62 (50.40%)	Fixed-term contracts	85 (69.10%)
Women	61 (49.60%)	Open-ended contracts	28 (22.76%)
**Relationship status**, *N*		Interim contracts	7 (5.70%)
Couple	88 (71.55%)	“Did not state”	3 (2.44%)
Single	25 (20.32%)	**Employment status**, *N*	
“Did not state”	10 (8.13%)	Employees	79 (64.23%)
**Language**, *N*		Blue-collar workers	28 (22.76%)
French	62 (50.40%)	Managers	12 (9.76%)
Dutch	61 (49.60%)	Self-employed	1 (0.81%)
**Sector**, *N*		“Did not state”	3 (2.44%)
Private	97 (78.86%)	**Working time**, *N*	
Public	25 (20.33%)	Full-time	40 (32.52%)
“Did not state”	1 (0.81%)	Part-time	3 (2.44%)
**Organization size**, *N*		“Did not state”	80 (65.04%)
<20	13 (10.57%)	**Work disability**, *N*	
20–49	3 (2.44%)	Yes	58 (47.15%)
50–250	18 (14.64%)	No	65 (52.85%)
>250	81 (65.85%)		
“Did not state”	8 (6.50%)		

* SEM: standard error of the mean. The bold is used to identify more easily each socio-demographic variable.

**Table 3:** Theoretical table to test the validity of the Oldenburg burnout inventory (OLBI).

		Method Tested	
		Positive OLBI	Negative OLBI
Reference method	Positive clinical judgement/EDTB	True Positive (TP)	False Negative (FN)
	Negative clinical judgement/EDTB	False Positive (FP)	True Negative (TN)

**Table 4:** Theoretical table to test the validity of the early detection tool of burnout (EDTB).

		Reference Method	
		Positive OLBI	Negative OLBI
Method tested	Positive clinical judgement/EDTB	True Positive (TP)	False Positive (FP)
	Negative clinical judgement/EDTB	False Negative (FN)	True Negative (TN)

**Table 5:** Cross-distribution of burnout diagnoses (*N*, %).

	Positive OLBI	Negative OLBI	
Positive clinical judgement/EDTB	Gr1 52 (42.27%)	Gr3 22 (17.89%)	74
Negative clinical judgement/EDTB	Gr2 16 (13.01%)	Gr4 33 (26.83%)	49
	68	55	123

**Table 6:** Comparison between test validity indicators of the EDTB and the OLBI.

Test Validity Indicators	Tested Methods	
	EDTB	OLBI
Sensitivity	0.76 *	0.70 *
Specificity	0.60	0.67
Positive predictive value	0.70	0.76
Negative predictive value	0.67	0.60
Accuracy	0.69	0.69

Note. * *p* < 0.05.

**Table 7:** Distribution of burnout diagnoses for occupational physicians (OPs) (*N*, %).

	Positive OLBI	Negative OLBI	
Positive clinical judgement/EDTB	39 (39%)	15 (15%)	54
Negative clinical judgement/EDTB	14 (14%)	32 (32%)	46
	53	47	100

**Table 8:** Distribution of burnout diagnoses for general practitioners (GPs) (*N*, %).

	Positive OLBI	Negative OLBI	
Positive clinical judgement/EDTB	14 (60.86%)	6 (26.08%)	20
Negative clinical judgement/EDTB	2 (0.08%)	1 (0.04%)	3
	16	7	23

**Table 9:** Comparison between the EDTB and the OLBI among OPs and GPs.

	All Physicians		Occ. Phys. (OPs)		Gen. Pract. (GPs)	
Method Tested	EDTB	OLBI	EDTB	OLBI	EDTB	OLBI
Sensitivity	0.76 *	0.70 *	0.74 *	0.72 *	0.87 *	0.70 *
Specificity	0.60	0.67	0.68 *	0.70 *	0.14	0.33
Positive predictive value	0.70	0.76	0.72	0.74	0.70	0.87
Negative predictive value	0.67	0.60	0.70	0.68	0.33	0.14
Accuracy	0.69	0.69	0.71	0.71	0.65	0.65

Note. * *p* < 0.05.

## Data Availability

The raw dataset is conserved by the authors from the University of Liège. The present study covered the measures from a study conducted in 2019 by Hansez, Rusu, Firket and Braeckman [26]. The research questions tested in the present study have not been investigated in previous work using this dataset.

## References

[1] FPS Employment, Labour and Social Dialogue Maladies Professionnelles. https://www.beswic.be/fr/themes/information-pour-les-medecins-traitants/maladies-professionnelles.

[2] National Institute for Health and Disability Insurance Incapacité de Travail de Longue Durée: Combien de Burnouts et de Dépressions de Longue Durée? Quel Coût Pour L’assurance Indemnités?. https://www.riziv.fgov.be/fr/statistiques/indemnites/Pages/incapacite-travail-longue-duree-combien-burn-outs-depressions.aspx#Une_augmentation_de_39,23_%_de_burnouts_et_de_d%C3%A9pressionde_longue_dur%C3%A9e_en_4_ans.

[3] Heureux P. (2018). Le burnout à la consultation du médecin généraliste. Rev. Sect. Sci. St. De L’ucl.

[4] Guseva Canu I., Mesot O., Györkös C., Mediouni Z. (2019). Burnout Syndrome in Europe: Towards a Harmonized Approach in Occupational Health Practice and Research. Ind. Health.

[5] World Health Organization Burn-Out An “Occupational Phenomenon”: International Classification of Diseases. https://www.who.int/news/item/28-05-2019-burn-out-an-occupational-phenomenon-international-classification-of-diseases.

[6] Bravo D.M., Suárez-Falcón J.C., Bianchi J.M., Segura-Vargas M.A., Ruiz F.J. (2021). Psychometric Properties and Measurement Invariance of the Maslach Burnout Inventory–General Survey in Colombia. Int. J. Environ. Res. Public Health.

[7] Schaufeli W.B., Desart S., De Witte H. (2020). Burnout Assessment Tool (BAT)—development, validity, and reliability. Int. J. Environ. Res. Public Health.

[8] Guseva Canu I., Marca S., Dell’Oro F., Balázs Á., Bergamaschi E., Besse C., Bianchi R., Bislimovska J., Koscec Bjelajac A., Bugge M. (2020). Harmonized Definition of Occupational Burnout: A Systematic Review, Semantic Analysis, And Delphi Consensus In 29 Countries. Scand. J. Work. Environ. Health.

[9] Hansez I., Firket P., Leclercq C. Stades du Burnout et le Type de Prévention Approprié Selon le Stade. Presented at the “Burnout: Les Clefs Pour Agir”, Bruxelles, Belgium, 12 December 2019. https://www.health.belgium.be/fr/stades-du-burnout-et-le-type-de-prevention-approprie-selon-le-stade.

[10] Demerouti E., Bakker A.B., Vardakou I., Kantas A. (2003). The convergent validity of two burnout instruments: A multitrait-multimethod analysis. Eur. J. Psychol. Assess..

[11] Guidetti G., Viotti S., Gil-Monte P., Converso D. (2018). Feeling Guilty or Not Guilty. Identifying Burnout Profiles Among Italian Teachers. Curr. Psychol..

[12] Shoman Y., Marca S.C., Bianchi R., Godderis L., van der Molen H.F., Guseva-Canu I. (2021). Psychometric properties of burnout measures: A systematic review. Epidemiol. Psychiatr. Sci..

[13] Abeltina M., Stokenberga I., Skudra J., Rascevska M., Kolesovs A. (2020). Burnout Clinical Subtypes Questionnaire (BCSQ-36): Reliability and validity study in Latvia. Psychol. Health Med..

[14] Schilling R., Colledge F., Brand S., Ludyga S., Gerber M. (2019). Psychometric properties and convergent validity of the Shirom–Melamed burnout measure in two German-speaking samples of adult workers and police officers. Front. Psychiatry.

[15] Schaufeli W.B., De Witte H., Desart S. (2020). Handleiding Burnout Assessment BPSEWS Tool (BAT).

[16] Schaufeli W.B., De Witte H., Desart S. (2019). Manual of Burnout Assessment Tool (BAT).

[17] Sakakibara K., Shimazu A., Toyama H., Schaufeli W.B. (2020). Validation of the Japanese Version of the Burnout Assessment Tool. Front. Psychol..

[18] Consiglio C., Mazzetti G., Schaufeli W.B. (2021). Psychometric Properties of the Italian Version of the Burnout Assessment Tool (BAT). Int. J. Environ. Res. Public Health.

[19] Schaufeli W.B., van Dierendonk D. (2000). Utrechtse Burnout Schaal (UBOS) Handleiding (Utrecht Burnout Scale Manual).

[20] Rotenstein L., Torre M., Ramos M., Rosales R., Guille C., Sen S., Mata D. (2018). Prevalence of Burnout Among Physicians. J. Am. Med Assoc..

[21] Qiao H., Schaufeli W. (2010). The Convergent Validity of Four Burnout Measures in A Chinese Sample: A Confirmatory Factor-Analytic Approach. Appl. Psychol..

[22] Sinval J., Queirós C., Pasian S., Marôco J. (2019). Transcultural Adaptation of the Oldenburg Burnout Inventory (OLBI) for Brazil and Portugal. Front. Psychol..

[23] Grove W., Zald D., Lebow B., Snitz B., Nelson C. (2000). Clinical Versus Mechanical Prediction: A Meta-Analysis. Psychol. Assess..

[24] Bell I., Mellor D. (2009). Clinical Judgements: Research and Practice. Aust. Psychol..

[25] Hansez I., Mairiaux P., Firket P., Braeckman L. (2010). Recherche Sur le Burnout au Sein de La Population Active Belge: Rapport Final [Research on Burnout in the Active Belgian Population: Final Report].

[26] Hansez I., Rusu D., Firket P., Braeckman L. (2019). Evolution 2010–2018 du Burnout en Belgique et Intérêt de L’utilisation Conjointe de Deux Outils de Diagnostic.

[27] FPS Employment, Labour and Social Dialogue Détection Précoce du Burnout: Outil Pour le Médecin Généraliste Burnout. https://emploi.belgique.be/fr/publications/detection-precoce-du-burnout-outil-pour-le-medecin-generaliste.

[28] Nahavandi K.H. (2018). Calculating Sensitivity, Specificity and Predictive Values for Medical Diagnostic Tests. Gene Cell Tissue.

[29] Hajian-Tilaki K. (2018). The choice of methods in determining the optimal cut-off value for quantitative diagnostic test evaluation. Stat. Methods Med. Res..

[30] Trevethan R. (2017). Sensitivity, Specificity, And Predictive Values: Foundations, Pliabilities, and Pitfalls in Research and Practice. Front. Public Health.

[31] Ægisdóttir S., White M., Spengler P., Maugherman A., Anderson L., Cook R., Nichols C., Lampropoulos G., Walker B., Cohen G. (2006). The Meta-Analysis of Clinical Judgment Project: Fifty-Six Years of Accumulated Research on Clinical Versus Statistical Prediction. Couns. Psychol..

[32] van Vugt E., Asscher J., Hendriks J., Stams G., Bijleveld C., van der Laan P. (2011). Assessment of Moral Judgment and Empathy in Young Sex Offenders. Int. J. Offender Ther. Comp. Criminol..

[33] Kirkhus L., Šaltytė Benth J., Rostoft S., Grønberg B., Hjermstad M., Selbæk G., Wyller T., Harneshaug M., Jordhøy M. (2017). Geriatric Assessment Is Superior to Oncologists’ Clinical Judgement in Identifying Frailty. Br. J. Cancer.

[34] Barroso N., Alcântara P., Botelho A., Douglas-de-Oliveira D., Gonçalves P., Flecha O. (2019). Prevalence of Self-Reported Versus Diagnosed Dentinal Hypersensitivity: A Cross-Sectional Study and ROC Curve Analysis. Acta Odontol. Scand..

[35] Schaufeli W.B., Bakker A.B., Hoogduin K., Schaap C., Kladler A. (2001). On the clinical validity of the Maslach Burnout Inventory and the Burnout Measure. Psychol. Health.

[36] Kleijweg J., Verbraak M., Van Dijk M. (2013). The Clinical Utility of The Maslach Burnout Inventory in A Clinical Population. Psychol. Assess..

[37] Wickramasinghe N., Dissanayake D., Abeywardena G. (2018). Clinical Validity and Diagnostic Accuracy Of The Maslach Burnout Inventory-Student Survey In Sri Lanka. Health Qual. Life Outcomes.

[38] Evans S., Reed G., Roberts M., Esparza P., Watts A., Correia J., Ritchie P., Maj M., Saxena S. (2013). Psychologists’ Perspectives on The Diagnostic Classification of Mental Disorders: Results from the WHO-Iupsys Global Survey. Int. J. Psychol..

[39] Bakker A.B., Demerouti E. (2017). Job demands–resources theory: Taking stock and looking forward. J. Occup. Health Psy-Chology.

[40] Hansez I., Laurent J. (2018). Création de Normes Pour la Version Francophone de L’outil « OLBI ».

[41] R Development Core Team (2021). R: A Language and Environment for Statistical Computing.

[42] Yang S., Berdine G. (2017). The receiver operating characteristic (ROC) curve. Southwest Respir. Crit. Care Chron..

[43] Landis J.R., Koch G.G. (1977). An application of hierarchical kappa-type statistics in the assessment of majority agreement among multiple observers. Biometrics.

[44] Power M., Fell G., Wright M. (2012). Principles for High-Quality, High-Value Testing. Evid. Based Med..

[45] Mbanga C., Makebe H., Tim D., Fonkou S., Toukam L., Njim T. (2018). Determinants of burnout syndrome among nurses in Cameroon. BMC Res. Notes.

[46] Nguyen Huynh A., Besse C., Mediouni Z., El May E., Shoman Y., Hansez I., Guseva Canu I. (2021). Diagnostic Performances of an Occupational Burnout Detection Method Designed for Healthcare Professionals. Int. J. Environ. Res. Public Health.

[47] Nguyen Huynh A., Béguelin A., Krief P., Marion-Veyron R., Mediouni Z., Regamey F., Staeger P., Guseva Canu I. (2021). Repérage et prise en charge des patients en burnout par les médecins d’Unisanté [Detection and treatment of burnout by physicians of Unisanté]. Rev. Med. Suisse.

[48] Salvagioni D., Melanda F., Mesas A., González A., Gabani F., Andrade S. (2017). Physical, Psychological and Occupational Consequences Of Job Burnout: A Systematic Review Of Prospective Studies. PLoS ONE.

[49] Fedris Projet Pilote Burn-Out. https://fedris.be/fr/node/2540.

[50] García-Arroyo J., Osca Segovia A. (2018). Effect sizes and cut-off points: A meta-analytical review of burnout in Latin American countries. Psychol. Health Med..

